# The Development of the Bacterial Community of Brown Trout (*Salmo trutta*) during Ontogeny

**DOI:** 10.3390/microorganisms11010211

**Published:** 2023-01-14

**Authors:** Katharina Keiz, Sebastian Ulrich, Jasmin Wenderlein, Patrick Keferloher, Anna Wiesinger, Klaus Neuhaus, Ilias Lagkouvardos, Helmut Wedekind, Reinhard K. Straubinger

**Affiliations:** 1 Institute of Infectious Diseases and Zoonosis, Department of Veterinary Sciences, Faculty of Veterinary Medicine, LMU Munich, Veterinärstr. 13, 80539 Munich, Germany; 2 Bavarian State Research Center for Agriculture (LfL), Institute for Fisheries (IFI), Weilheimer Straße 8, 82319 Starnberg, Germany; 3 Core Facility Microbiome, ZIEL—Institute for Food & Health, Technical University of Munich, Weihenstephaner Berg 3, 85354 Freising, Germany; 4 Hellenic Centre for Marine Research (HCMR), Institute of Marine Biology and Aquaculture (IMBBC), 715 00 Heraklion, Greece

**Keywords:** fish, brown trout, *Salmo trutta*, development, ontogeny, microbiome, bacterial community

## Abstract

Brown trout (*Salmo trutta*) is an important aquaculture species in Germany, but its production faces challenges due to global warming and a high embryo mortality. Climate factors might influence the fish’s bacterial community (BC) and thus increase embryo mortality. Yet, knowledge of the physiological BC during ontogeny in general is scarce. In this project, the BC of brown trout has been investigated in a period from unfertilized egg to 95 days post fertilization (dpf) using 16S rRNA gene amplicon sequencing. Developmental changes differed between early and late ontogeny and major differences in BC occurred especially during early developmental stages. Thus, analysis was conducted separately for 0 to 67 dpf and from 67 to 95 dpf. All analyzed stages were sampled in toto to avoid bias due to different sampling methods in different developmental stages. The most abundant phylum in the BC of all developmental stages was Pseudomonadota, while only two families (Comamonadaceae and Moraxellaceae) occurred in all developmental stages. The early developmental stages until 67 dpf displayed greater shifts in their BC regarding bacterial richness, microbial diversity, and taxonomic composition. Thereafter, in the fry stages, the BC seemed to stabilize and changes were moderate. In future studies, a reduction in the sampling time frames during early development, an increase in sampling numbers, and an attempt for biological reproduction in order to characterize the causes of these variations is recommended.

## 1. Introduction

A fast-growing world population goes hand in hand with an increasing demand for high-quality protein sources [1]. Between 1961 and 2017, fish consumption increased at an average annual rate of 2.4% to meet this need [2]. Consequently, global capture and aquaculture fisheries both reached record production in 2018 [3]. Further rises in fish production cannot be expected, as fishing quotas can no longer be increased considerably in the future as the global fish stocks are already in a critical condition and declining [4]. Therefore, the importance of aquaculture will further increase. Salmonids (Salmonidae) such as salmon and trout (both *Salmo* and *Oncorhynchus* species) are popular food fish and a good source of high-quality animal protein [5,6]. Many salmonid species are raised in large net pens in coastal areas. These marine farming methods lead to some ecological and environmental problems, such as the unfiltered emission of drugs and feces into the surrounding water [7]. The contamination of water is worrying, especially when One Health concerns are considered, as feces might contain pathogens and the use of antibiotics might lead to the spread of resistance genes and resistant bacteria via the water [8]. Further, the maintenance of clean water is goal number six of the UN Sustainable development goals [9] and must be taken seriously. Thus inland fish production of trout in small pond-based fish farms with filtration systems and sedimentation of suspended solids is likely the more ecological option [10]. Another advantage of local fish production is its excellent ecological performance by eliminating long transport routes [11]. The brown trout (*Salmo trutta*) is native to Eurasia and North Africa [12]. In Germany, the farming of salmonids is practiced mostly in earthen or concrete ponds, managed as flow-through systems. The production of brown trout is used for human consumption, as well as for stocking purposes.

Even though inland trout aquaculture has a long tradition, it faces various challenges due to climate change. Increasing water temperatures and periods of low runoff limit the availability of cold, oxygen-rich water needed in salmonid production [13,14,15]. However, suboptimal water quality parameters affect the fish’s fitness negatively and alter the microbiome of aquatic organisms often detrimentally [16,17,18]. In the future, these obstacles and limitations must be overcome to maintain or increase output. Thus, to benefit or even increase fish health and fitness the manipulation of the bacterial community (BC), especially in early life stages, might be an option. The BC comprise all bacterial members of the microbiome, which itself is defined as the entirety of all microorganisms (bacteria, archaea, lower and higher eukaryotes, and viruses), their genomes, and the environmental conditions surrounding them in a given habitat [19]. The BC within a host organism is essential for immune defense and vertebrate health [20,21]. Therefore, a dysbiosis of the BC due to changed environmental conditions increases the fish’s susceptibility to infection [22,23]. As fish are surrounded by water and microorganisms during their entire lifetime [24], they are directly and immediately confronted with the microbiome in their habitat to a greater extent than terrestrial organisms. Thus, embryonic stages are under strong pathogen pressure and the egg’s surface additionally provides a suitable substrate for bacterial growth [25,26]. It has been suggested that the BC on the embryo’s surface influences embryonic death [18]. Understanding the microbiome’s development during rearing is essential to provide optimal conditions after hatching when organs of the immune system develop [27]. The development of the microbiome and its balance is complex in a fish farm, where many different environmental factors, e.g., water temperature [28], diet [29], and handling [30], influence the microbiome. Further, stress factors (i.e., environmental or populational) to which the fish is exposed in early developmental stages can have long-lasting effects on the BC [31]. A poor health status in some aquaculture facilities is an economic problem and can limit the number of fish produced.

The brown trout is one of the economically most important freshwater salmonids produced in Bavaria, Germany [32]. Knowledge of the composition of the microbiome of brown trout is scarce, especially considering the early developmental stages. Existing research focuses mainly on the vegetarian and thus resource-friendly feeding of brown trout and other salmonids [33,34]. The BCs investigated in other studies often were only analyzed in view of higher taxa level. Even at the phylum level, significant differences between several studies are apparent, each containing divergent results regarding the most dominant phyla [23,35,36]. The basis for possible intervention pathways regarding the BC, with a focal point on the prevention of diseases, is a comprehensive knowledge of the typical microbiota of the brown trout during embryonal and early development. Therefore, the aim of the present study was the description of the development of the BC during ontogeny. Using 16S rRNA gene amplicon sequencing, we characterized the BC of the whole fish from unfertilized egg to fry and compared these life stages.

## 2. Materials and Methods

### 2.1. Fish Management

All experiments involving brown trout were conducted in compliance with the requirements of the German Animal Welfare Act [37]. The fish were reared at Bavarian State Research Center for Agriculture, Institute for Fisheries (Starnberg, Germany). The use of animals did not demand governmental approval as the animals were reared in a commercial and scientific fish farm. During the experiment, no harmful interventions were conducted. Fish were narcotized and then killed using decapitation according to the Council Regulation (EC) No. 1099/2009 and the German *Tierschutz-Schlachtverordnung* (Animal Welfare Slaughter Ordinance) [38,39].

The parental fish originated from the Institute’s breeding stock, which is kept for research purposes. In January 2020, a total of 20 milters and 20 spawners were stripped on two dates with a time difference of 14 days (line A and line B). Every female was crossed with several males to increase the genetic diversity of the experimental fish. Therefore, eggs from the same spawner were fertilized with sperm of different milters from the same population. The brown trout of the two lines entered the different developmental stages at different timepoints and were sampled accordingly with a time delay of 14 days.

After fertilization, the eggs were placed in horizontal breeding troughs with egg incubation baskets, ensuring a constant flow of degassed and aerated spring water. The brown trout eggs from line A (approximately 30,000) were randomly distributed among two commercial hatching troughs, while the comparatively fewer brown trout eggs from line B (approximately 20,000) fitted into a single trough. The incubation baskets were further used for start-feeding the swim-up fry after the yolk sac had been absorbed. Afterward, the incubation baskets were removed, and the fry were kept directly in the troughs. Temperature, pH, oxygen content, and conductivity were measured on every sampling day (mean temperature = 10.21 ± 0.14 °C, mean pH = 7.67 ± 0.03, mean oxygen = 12.99 ± 0.49 mg/L, mean conductivity = 799 ± 5 μS/cm).

The brown trout larvae hatched 39 to 40 days post fertilization (dpf). Feeding was initiated at 67 dpf. The fish were then hand-fed several times a day to satiety, using a dry commercial diet (INICIO Plus, BioMar A/S, Brande, Denmark).

To describe the development of the microbiome during ontogeny, six samples from line A and five samples from B were collected from the following developmental stages: unfertilized eggs (EGG, 0 dpf), eyed eggs (EYG, 35 dpf), sac fry (SCF, 48 dpf), and swim-up fry (SUF, 67 dpf). Furthermore, five samples were collected from the older fry stages of both lines on 69 dpf (FY1), 74 dpf (FY2), 82 dpf (FY3), 88 dpf (FY4), and 95 dpf (FY5). The total duration of the trial was 95 days.

### 2.2. Post-Mortem Sampling

In total, 99 samples were collected from all developmental stages for microbiome analysis, whereby 56 samples were available for the final analysis. SCF, SUF, and FY1-FY5 stages were narcotized and immediately killed by decapitation [38]. Due to size limitations and the lack of differentiation of the gastrointestinal tract before first feeding the samples from SCF and SUF were transferred *in toto* into a 2.0 mL reaction tube (Eppendorf AG, Hamburg, Germany) prefilled with 600 µL of Stool DNA Stabilizer (Invitek Molecular GmbH, Berlin, Germany). The tubes were flash-frozen in liquid nitrogen and stored at −80 °C. To avoid biases due to the sampling method, the same procedure using the entire fish was carried out with the stages FY1-FY5, without differentiation of different body parts and organs such as the intestines. EGG and EYG were immediately transferred *in toto* to a 2.0 mL reaction tube (Eppendorf AG) prefilled with 600 µL of DNA-Stool Stabilizer (Invitek Molecular GmbH) and flash-frozen in liquid nitrogen and stored at −80 °C.

When sampling for this experiment was finished, all samples were transferred on dry ice to the ZIEL—Institute for Food & Health of the Technical University of Munich for analysis.

### 2.3. Metagenomic DNA Extraction

Metagenomic DNA was extracted according to a modified protocol by Godon et al. (1997) [40] as described previously by Reitmeier et al. (2021) [41]. Briefly, the complete material was transferred into 2.0 mL lysing matrix B tubes (MP Biomedicals GmbH, Eschwege, Germany), prefilled with 0.1 mm silica beads. After adding guanidinium thiocyanate and N-lauroylsarcosine, samples were homogenized with a FastPrep-24^®^ (MP Biomedicals GmbH) using three cycles (40 s, 6.5 m/s) and cooling with dry ice. Then, polyvinylpyrrolidone was added and after centrifuging the supernatant was treated with RNase A. DNA was cleaned up using columns and stored at −20 °C until further processing.

### 2.4. 16S rRNA Gene Amplicon Sequencing

The composition of the BC of the complete fish at different developmental stages was assessed by high throughput 16S rRNA gene amplicon sequencing of the V3/V4-regions at Eurofins Genomics Germany GmbH (Ebersberg, Germany). The amplification was conducted via a two-step PCR using primers 341F and 785R [42]. The first PCR amplified the V-regions of the 16S rRNA target gene. A sample-unique barcode and Illumina sequencing adaptors were added to both ends in the second PCR to each amplicon. The DNA concentrations were measured and adjusted to the required DNA quantity. Pooled samples were sequenced, using reagent kit v3, in paired-end mode (2 × 300 bp) in a MiSeq (Illumina, San Diego, CA, USA).

### 2.5. Data Processing

The 16S rRNA gene amplicon data were analyzed as described previously [43]. Briefly, at first, all fastq files were processed using the “Integrated Microbial Next-Generation Sequencing” (IMNGS) pipeline [44], a UPARSE-based platform [45]. Sequences were filtered (quality trimming score of less than 15) and paired. Paired reads with expected errors of less than two and sequences with a length smaller than 300 and higher than 600 nucleotides were excluded. Further, remaining reads were trimmed for ten nucleotides on each end, removing primers partially. The presence of chimeras was tested with UCHIME [46]. Following the first processing on the IMNGS platform, samples with a read count less than 100 and non-sufficient quality observed in rarefaction curves have been excluded from further analysis. Thus, secondly, remaining data were re-submitted to IMNGS pipeline for the final analysis. The most recent version of IMNGS supplies operational taxonomic units (OTUs) and denoised zero-radius operational-taxonomic units (zOTUs) for different parts of the analysis. OTUs were clustered at 97% sequence similarity [44], while zOTUs were calculated using UNOISE 2 [47] from the USEARCH 11 package [48]. Only those OTUs and zOTUs occurring with at least 0.25% relative abundance in at least one sample were kept for further analysis. Taxonomy was assigned at an 80% confidence level with SILVA [49].

Afterward, the data provided by IMNGS was refined by manually editing the automated phylogenetic tree and taxonomy using SILVA [49]. Sequences were re-aligned and improved phylogenetic trees were afterwards constructed using the neighbor-joining method available on the software MEGA X [50]. Taxonomy was checked using the 16S-based ID provided by EzBioCloud and, if necessary, adapted [51]. Further, the taxonomy was refined according to the nomenclature provided by the LPSN database [52] using the phylum names introduced in 2021 [53]. Since this leads to a renaming of all phyla occurring in this study, the former names are given in parentheses (at the first mention) for better comparability with older studies.

All downstream analyses were carried out using Rhea [54], a modular pipeline for microbial profiling of 16S rRNA gene amplicon sequencing data in an R programming environment (R 4.1.2, R Foundation for Statistical Computing, Vienna, Austria) as described previously [54]. The pipeline is available on the GitHub repository (https://github.com/Lagkouvardos/Rhea, accessed on 7 September 2022). The OTU and zOTU tables of all experimental samples can be found in the Supplementary Material (Tables S1 and S2). For the α-diversity, the effective richness and the effective Shannon diversity were calculated using OTUs [55]. All further parameters were calculated using zOTUs. Based on generalized UniFrac distances, β-diversity was calculated [56]. The Benjamini-Hochberg method was used for correcting *p* values for multiple testing [57]. For statistical testing, taxa with a prevalence equal to or more than 20% (proportion of samples positive for the respective taxa) in at least one of the groups and a relative abundance of equal to or more than 0.25% were considered. For multiple groups, a Kruskal–Wallis rank-sum test was applied. Subsequently, the Wilcoxon rank-sum test was used for pairwise comparisons. A non-linear Fisher’s exact test was used to determine the differences between samples with a low prevalence.

Data were visualized using Illustrator CS6 version 16.0.0 (Adobe Inc., San José, CA, USA). The most abundant taxa were visualized using the software Prism, version 2010 (GraphPad Software, San Diego, CA, USA). Venn diagrams were created using Origin Pro version 2021 (OriginLab Corporation, Northampton, MA, USA).

## 3. Results

An average of 11,858 sequences (SD = 9065) per sample was obtained from the 16S rRNA gene amplification, accounting for 664,052 sequences in total. A total of 515 molecular species were found at the OTU level (operational taxonomic unit), while the analysis at the zOTU level (zero-radius operational taxonomic unit; molecular strains) yielded 886 zOTUs [58].

**Figure 1:**
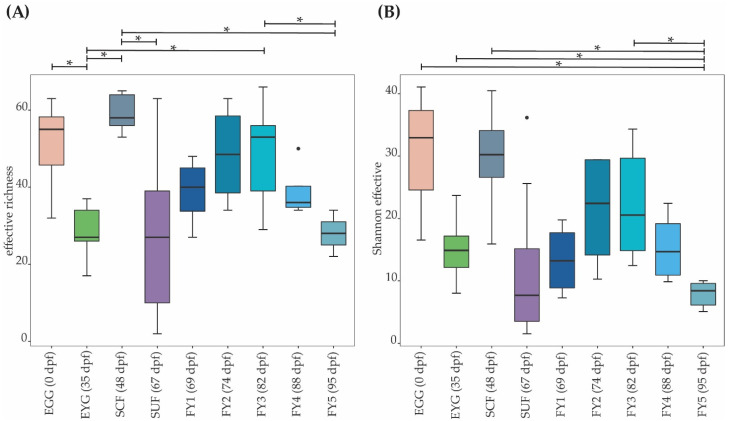
α-diversity shown as effective richness (**A**) and Shannon effective diversity (**B**) of the different developmental stages of brown trout. Boxplots depict the median (thick bar), upper and lower quartile (within the box), and standard deviation (whiskers). Brackets above the individual boxplots indicate significance for pairwise comparison. Outliers are shown as dots. EGG, egg; EYG, eyed egg; SCF, sac fry; SUF, swim-up fry; FY1, fry 69 dpf; FY2, fry 74 dpf; FY3, fry 82 dpf; FY4, fry 88 dpf; FY5, fry 95 dpf; dpf, days post fertilization. *p* value summary: * *p* ≤ 0.05.

α-diversity was analyzed at the OTU level, considering effective richness and Shannon effective diversity (Table 1, Figure 1). Samples EGG and SCF displayed the highest α-diversity parameters. Interestingly, in the EYG stages, a lower effective richness and Shannon effective diversity was observed compared to the developmental stages before and after EYG (i.e., EGG and SCF). The difference in effective richness was significant between EYG and the stages EGG, SCF, and FY3. Furthermore, the effective richness for SUF displayed the highest overall distribution. Species diversity initially increased from SUF to FY3. After reaching the highest effective richness of the late developmental stages in FY3, species diversity decreased until the last sampling time point in stage FY5, with a significant difference between FY3 and FY5 (*p* ≤ 0.05).

**Figure 2:**
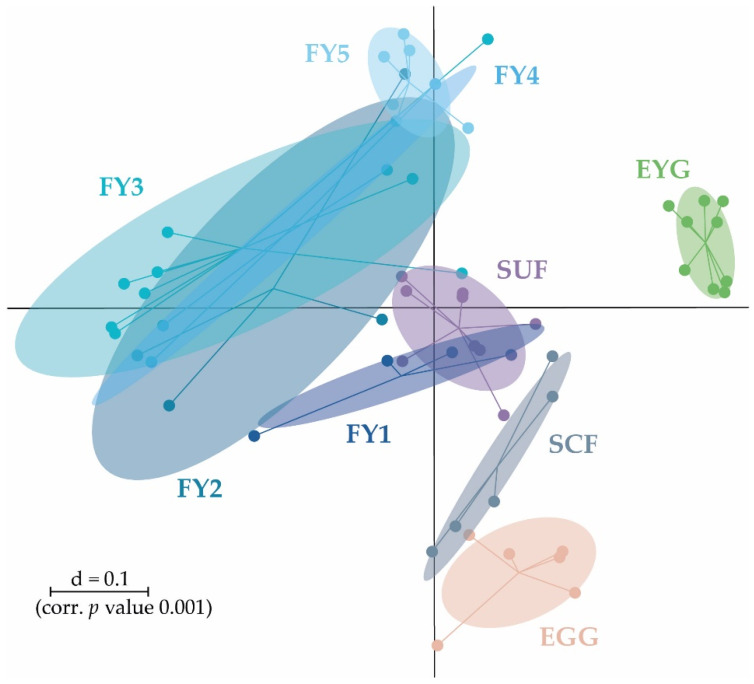
β-diversity of all samples shown as a multidimensional-scaling (MDS) plot. The scale is an indicator for the variation in the taxonomic makeup of microbiota between samples (β-diversity) based on generalized UniFrac distances (d = 0.1 marks a 10% difference). EGG, egg; EYG, eyed egg; SCF, sac fry; SUF, swim-up fry; FY1, fry 69 dpf; FY2, fry 74 dpf; FY3, fry 82 dpf; FY4, fry 88 dpf; FY5, fry 95 dpf; dpf, days post fertilization.

Microbial profiles in the early developmental stages from EGG to SUF seemingly displayed distinct clusters separated from each other and the fry stages (FY1–FY5). The three earliest stages EGG, EYG, and SCF seemed to cluster farthest from the other developmental stages. The cluster of the later developmental stages (i.e., FY1–FY5) display a higher overlap and wider intra-group distribution. SUF and FY1, the stages collected just before and two days after the first feeding, might have an overlap due to the short time interval between sampling. In general, with the exception of EYG, consecutive stages clustered closest (Figure 2).

### 3.1. Taxonomic Structure of Bacterial Communities (BC) of All Developmental Stages

#### 3.1.1. Description of the BC on Phylum Level

**Figure 3:**
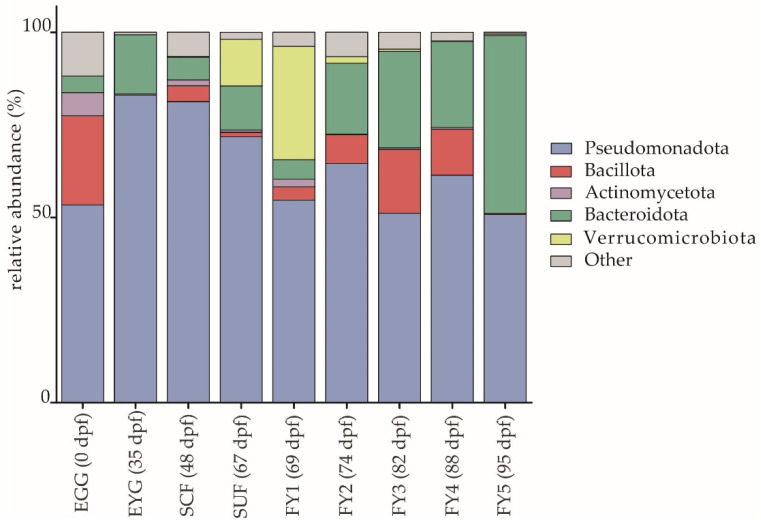
Relative abundances (%) of the five most abundant bacterial phyla in the BCs depicted for all stages. EGG, egg; EYG, eyed egg; SCF, sac fry; SUF, swim-up fry; FY1, fry 69 dpf; FY2, fry 74 dpf; FY3, fry 82 dpf; FY4, fry 88 dpf; FY5, fry 95 dpf; dpf, days post fertilization.

Pseudomonadota (formerly Proteobacteria) was the most abundant phylum in the BC of all developmental stages. The relative abundance varied between sampling times but always displayed a relative abundance of at least 50.0% (Figure 3). The different developmental stages displayed distinct changes even at the phylum level. These shifts were often caused by the increase or decrease of only one or a few families (e.g., Rubritaleaceae or Flavobacteriaceae). Bacteroidota (formerly Bacteroidetes) increased in the BC from the EGG to EYG stage, then alternated in the BC of SCF, SUF, and FY1 samples and strongly increased between FY2 and FY5. While Bacillota (previously Firmicutes) were highest in EGG samples, SCF (*p* ≤ 0.05 between EYG and SCF), SUF, and FY1 displayed lower relative abundances. Subsequently, Bacillota increased until FY3 (*p* ≤ 0.05 between EYG and FY3) and then decreased strongly until FY5. Interestingly, the phylum Verrucomicrobiota (formerly Verrucomicrobia) was observed at high abundances only in SUF (12.6%) and FY1 (30.6%), and at very low abundances (below 2.0%) in the FY2 and FY3 samples (Figure 3).

#### 3.1.2. Description of the BC on Family Level

**Figure 4:**
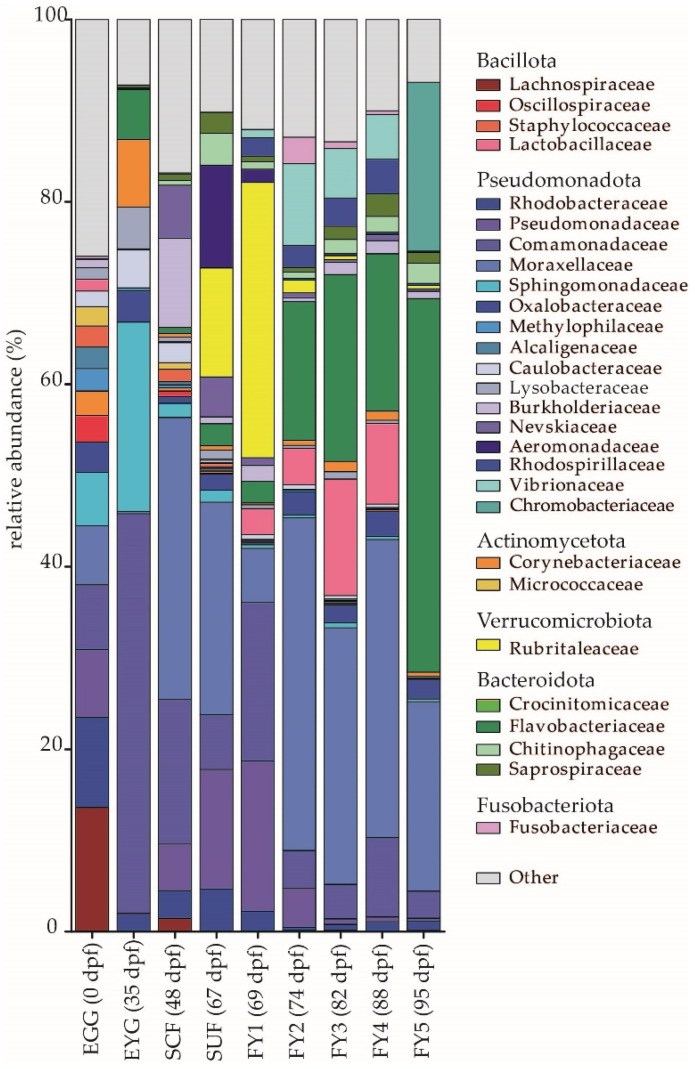
Bacterial families with a relative abundance of 2.0% or higher in at least one stage in the BC shown for all developmental stages. EGG, egg; EYG, eyed egg; SCF, sac fry; SUF, swim-up fry; FY1, fry 69 dpf; FY2, fry 74 dpf; FY3, fry 82 dpf; FY4, fry 88 dpf; FY5, fry 95 dpf; dpf, days post fertilization.

At the family level, the most abundant families were either Moraxellaceae, Comamonadaceae, Rubritaleaceae, or Flavobacteriaceae (Figure 4), although these families were distributed unevenly in the different developmental stages. The strongest shifts in the family composition occurred in the early developmental stages (i.e., EGG, EYG, SCF, and SUF), while the later developmental stages displayed a more similar family composition and most changes were observed in regard to the abundances of different families.

Interestingly, in the EGG samples, most of the occurring families were evenly represented. Here, the strongest family was Lachnospiraceae with a relative abundance of 13.6%, which could not be observed in any later stage. Following this, the families Rhodobacteraceae (9.9%), Pseudomonadaceae (7.4%), Comamonadaceae (7.1%), Moraxellaceae (6.5%), and Sphingomonadaceae (5.9%) were distributed evenly (Figure 4). Strong relative abundances of Lachnospiraceae explained the high number of the phylum Bacillota in the EGG stage, while in later stages high relative abundances of Bacillota mostly originated from the family Lactobacillaceae (i.e., FY2, FY3, and FY4). Further, the phylum Pseudomonadota was composed of different and relatively evenly distributed families (Figure 4; families colored in shades of blue) in the EGG, while in most other stages two to three families with a high relative abundance and few other families with a low relative abundance formed the phylum Pseudomonadota. High relative abundances of the families Sphingomonadaceae (20.8%), Crocinitomicaceae (7.4%), and Lysobacteriaceae (4.6%) were unique for the EYG stage. Furthermore, the EYG samples displayed the second highest relative abundance of a singular family (Comamonadaceae, 43.8%; Figure 4). Besides the above-mentioned families, EYG contained Flavobacteriaceae (5.5%) with an abundance above 5.0%. Moraxellaceae showed a high abundance in the SCF (30.9%) for the first time and remained at a high level except for FY1 (5.9%). Moraxellaceae was followed in abundance by Comamonadaceae (15.9%), and Burkholderiaceae (9.8%; Figure 4). SUF contained the families Moraxellaceae (23.3%), Pseudomonadaceae (13.1%), Rubritaleaceae (12.0%), and Aeromonadaceae (11.3%) in decreasing order (Figure 4). In this stage, Comamonadaceae decreased to 6.0% of its relative abundance (Figure 4). In the BC of the FY1 stage, the family Rubritaleaceae dominated with 30.2% at family level. Thereafter, Comamonadaceae (17.4%), Pseudomonadaceae (16.5%), and Moraxellaceae (5.9%) appeared with an abundance above 5.0%. The high number of Rubritaleaceae in this stage explains the high abundance of the phylum Verrucomicrobiota (Figure 4). Between FY1 and FY2, a strong shift in the arrangement of the families was observed. FY2 was dominated by Moraxellaceae (36.5%) and Flavobacteriaceae (15.2%), while Vibrionaceae (9.0%) was observed for the first time to have abundances above 1.0%. Pseudomonadaceae (4.3%) belonged to the most abundant families in the previous two stages but displayed a strong descent in FY2. In subsequent stages, Pseudomonadaceae was observed in abundances below 0.5% only (Figure 4). The familial composition of FY3 was mostly comparable to FY2. However, after Moraxellaceae (28.1%) and Flavobacteriaceae (20.5%), in this stage Lactobacillaceae (12.8%) appeared, followed by Vibrionaceae (5.4%). Similarly, FY4 was dominated by Moraxellaceae (32.7%), Flavobacteriaceae (17.3%), and Lactobacillaceae (8.9%). Then, Comamonadaceae (8.7%) and Vibrionaceae (4.9%) followed (Figure 4). In the subsequent FY5 stage, sampled only seven days after the FY4 stage, the composition shifted strongly. FY5 was dominated by Flavobacteriaceae (41.0%), which doubled in relative abundance in only one week, while Moraxellaceae (20.8%) decreased to the extent of a third. Another highly abundant family, Chromobacteriaceae (18.5%), was observed to have abundances above 0.006% for the first time in this stage (Figure 4).

### 3.2. Bacterial Communities (BC) of the Early Developmental Stages from Egg to Swim-Up Fry

The early and the late developmental stages were analyzed separately. For once, during early ontogeny the fish undergoes stronger developmental change, i.e., hatching, consumption of the yolk, resorption of the yolk sac, and mouth opening. While in comparison between fry stages, developmental changes are characterized by growth and maturation only.

**Figure 5:**
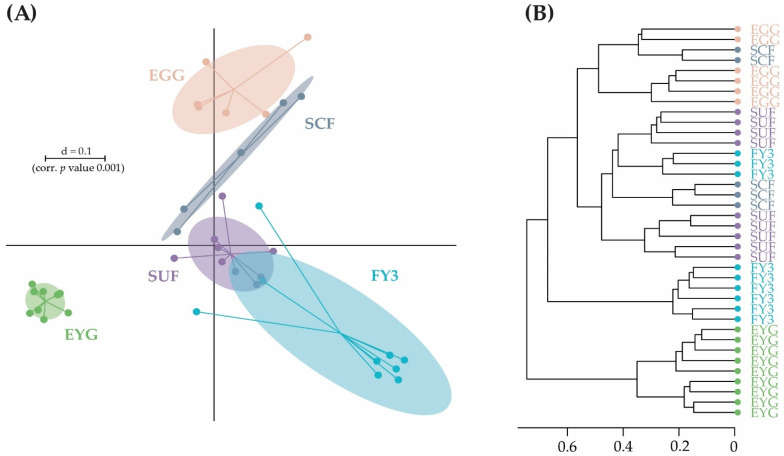
(**A**) β-diversity of early developmental stages. BC is shown as a multidimensional-scaling (MDS) plot. The scale is an indicator for the variation in the taxonomic makeup of microbiota between samples (β-diversity) based on generalized UniFrac distances (d = 0.1 marks a 10% difference). (**B**) Dendrogram of samples from early developmental stages based on wards clustering method (d = 0.1 marks a 10% difference). EGG, egg; EYG, eyed egg; SCF, sac fry; SUF, swim-up fry; FY3, fry 82 dpf; dpf, days post fertilization.

**Figure 6:**
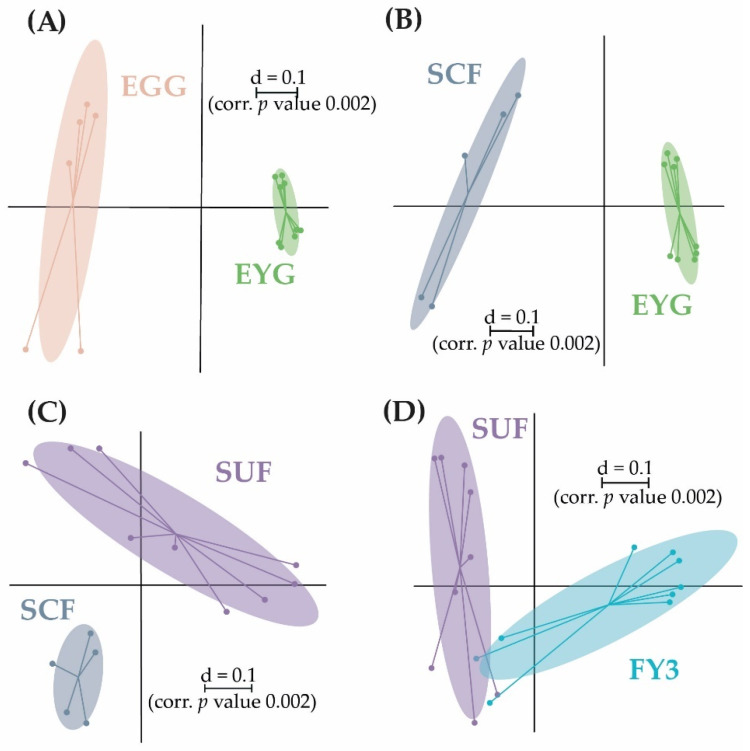
Pairwise β-diversity of the BC of the early developmental stages displayed as a multidimensional-scaling (MDS) plots. The scale is an indicator for the differences in the taxonomic makeup of microbiota between samples (β-diversity) based on generalized UniFrac distances (d = 0.1 marks a 10% difference). Pairwise β-diversity between EGG and EYG (**A**); EYG and SCF (**B**); SCF and SUF (**C**); SUF and FY3 (**D**). EGG, egg; EYG, eyed egg; SCF, sac fry; SUF, swim-up fry; FY3, fry 82 dpf; dpf, days post fertilization.

**Figure 7:**
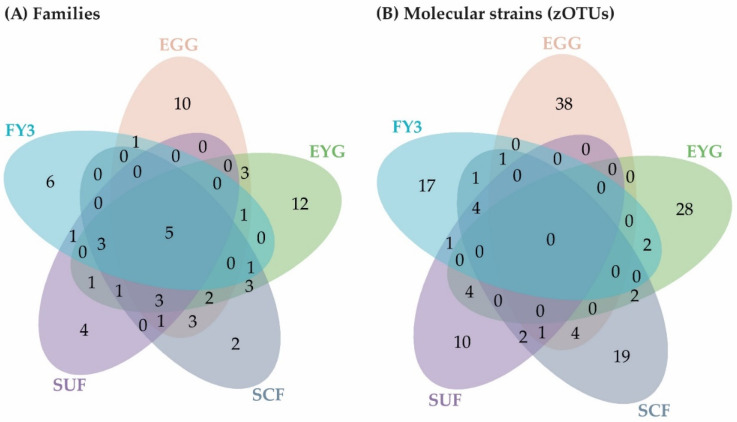
Venn diagram depicting unique and shared families (**A**) and molecular strains (zOTUs) (**B**) among the early developmental stages (EGG-SUF) and FY3 (cut-off: 0.5% mean relative abundance in the BC of each stage). EGG, egg; EYG, eyed egg; SCF, sac fry; SUF, swim-up fry; FY3, fry 82 dpf; dpf, days post fertilization.

The early developmental stages (i.e., EGG, EYG, SCF, SUF, and FY3) are characterized by strong changes in fish ontogeny, which were reflected in distinct shifts in the fish’s BC. The cluster of all stages, besides EYG, seemed closest to their preceding and subsequent sampling time point (Figure 5A). What was particularly striking was that the EYG clustered far away from the EGG and SCF, even though it is the intermediate stage. This is exceptionally clear in the dendrogram (Figure 5B). There was a significant difference between all the stages at the transition from one stage to the next (Figure 6). All developmental stages shared five families: Rhodobacteraceae, Comamonadaceae, Moraxellaceae, Lysobacteraceae, and Burkholderiaceae (Figure 7A). Many families and zOTUs seemed to emerge at one point but increase or even decrease thereafter, while others were observed in the EGG stage BC only. In the following paragraph, each stage is compared with its successor describing the changes in BC over the course of development.

**Figure 8:**
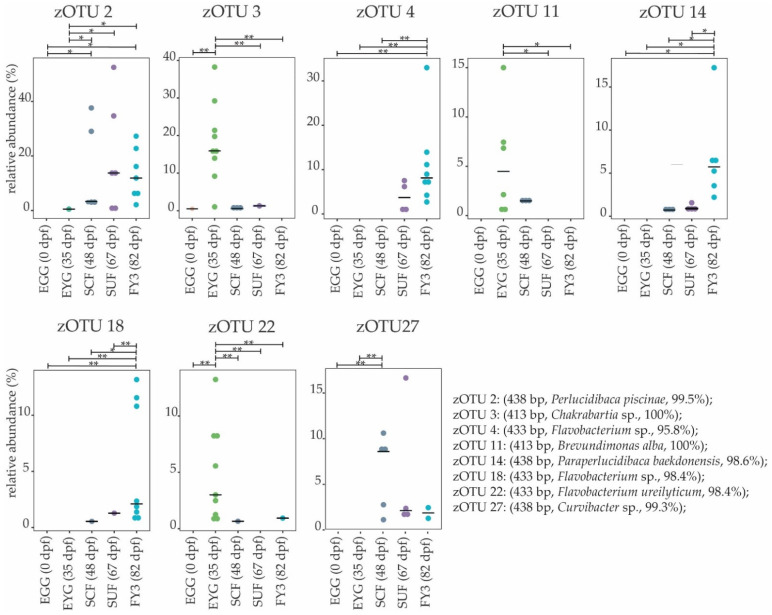
Molecular strains in the early developmental stages are shown. Boxplots depict the median (thick bar) and individual samples (points). Brackets above the individual boxplots indicate the significance level for pairwise comparison. zOTUs were identified by EzBiocloud; the sequence length, the closest relative taxon, and the sequence similarity score of zOTUs are shown in the order of appearance. EGG, egg; EYG, eyed egg; SCF, sac fry; SUF, swim-up fry; FY3, fry 82 dpf; dpf, days post fertilization. *p* value summary: * *p* ≤ 0.05, ** *p* ≤ 0.01.

EGG and EYG stages are unhatched embryonal stages with a time difference of 35 days. Between the EGG’s and EYG’s BC, effective richness decreased significantly (Figure 1A). The microbial profiles of these two stages displayed clearly separated clusters that differed significantly (Figure 6A). Here, different EGG samples seemed to cluster wider than EYG samples. The families and the molecular strains that were unique for one stage or shared among different stages are displayed in a Venn diagram (Figure 7). Both stages shared 14 families with a relative abundance higher than 0.5% (EGG 29 families, EYG 35 families; Figure 7A). Of these shared families, significant differences between the families Comamonadaceae (*p* ≤ 0.005), Corynebacteriaceae (*p* ≤ 0.05), Lachnospiraceae (*p* ≤ 0.05), and Moraxellaceae (*p* ≤ 0.05) were observed. The absence of the families Alcaligenaceae (*p* ≤ 0.05), Crocinitomicaceae (*p* ≤ 0.05), Flavobacteriaceae (*p* ≤ 0.001), Micrococcaceae (*p* ≤ 0.05), Staphylococcaceae (*p* ≤ 0.05), Streptococcaceae (*p* ≤ 0.05), and Pseudomonadaceae (*p* ≤ 0.01) was significant. Interestingly, EYG’s BC consisted of 35 families, but only 36 zOTUs, whereas the EGG’s BC consisted of 29 families and 44 zOTUs (Figure 7B). While there have been overlaps at the family level, no zOTUs were shared between these two stages (Figure 7B). On that account, significances at the zOTU level only arose due to the absence of species in either EGG or EYG (zOTU 20, *Hydrogenophaga taeniospiralis* with 99.8% similarity; zOTU 21, *Moraxella osloensis* with 100% similarity; zOTU 22, *Flavobacterium ureilyticum* with 99.8% similarity; zOTU 24, *Paucibacter oligotrophus* with 98.6% similarity; zOTU 39, *Curvibacter lanceolatus* with 97.7% similarity; zOTU 54, *Methylophilus quaylei* with 97.3% similarity; zOTU 72, *Alcaligenes aquatilis* with 99.5% similarity; and zOTU 81, *Rhizobacter profundi* with 99.5% similarity), while zOTU 3 (*Chakrabartia* species with 100% similarity) was significant due to its low abundance in EGG compared to EYG (Figure 8).

After the embryonal stages EGG and EYG, the SCF hatch. This stage is characterized by a closed mouth and the presence of a yolk sac. In comparison to the embryonal stage, where only the egg’s surface is exposed to the environment, in the sac fry stage the fish’s mucosa is in direct contact with the environment.

The effective richness increased significantly between EYG and SCF (Figure 1A). Microbial profiles of SCF displayed a more heterogenous cluster than EYG and both stages differed significantly in their β-diversity (Figure 6B). With a relative abundance of at least 0.5%, EYG contained 35 families and SCF 24 different families. These stages shared the highest number of families between all early developmental stages with 18 families (Figure 7A). Of these shared families, Comamonadaceae (*p* ≤ 0.005), Lachnospiraceae (*p* ≤ 0.05), and Moraxellaceae (*p* ≤ 0.05) differed significantly, while the absence of the families Burkholderiaceae (*p* ≤ 0.005), Flavobacteriaceae (*p* ≤ 0.05), Nevskiaceae (*p* ≤ 0.005), Pseudomonadaceae (*p* ≤ 0.005), and a family of the order Saprospirales (*p* ≤ 0.005) was statistically significant. Both stages displayed nearly the same number of zOTUs (EYG: 36; SCF: 34), whereby, they shared two zOTUs only (Figure 7B). As in the previous comparison, significant differences at the zOTU level were observed due to either the absence of a molecular strain (zOTU 20, *Hydrogenophaga taeniospiralis* with 99.8% similarity; zOTU 22, *Flavobacterium ureilyticum* with 99.8% similarity; zOTU 27, *Curvibacter* species with 99.3% similarity; zOTU 39, *Curvibacter lanceolatus* with 97.7% similarity; zOTU 49, *Limnobacter thiooxidans* with 100% similarity; zOTU 110, *Perlucidibaca piscinae* with 98.9% similarity; zOTU 119, *Agitococcus lubricus* with 96.5% similarity; zOTU 165, *Stagnimonas* species with 98.9% similarity; and zOTU 174, *Brevundimonas* species with 99.8% similarity) or a very low number of samples containing zOTU 2 (*Perlucidibaca piscinae* with 99.5% similarity; Figure 8). Many molecular strains appeared only in the EYG, examples named here appeared with a mean relative abundance of over 1.0% in at least 50% of the samples: zOTU 20 and zOTU 23 (both *Hydrogenophaga taeniospiralis* with 99.8% similarity), zOTU 24 (*Paucibacter digotrophus* with 98.6% similarity), zOTU 34 (*Rhodoferax aquaticus* with 99.3% similarity), zOTU 50 (*Rhodoferax* species with 98.4% similarity), zOTU 62 (*Rhodoferax bucti* with 99.3% similarity), and zOTU 82 (*Rhizobacter profundi* with 99.5% similarity).

The stage succeeding SUF is characterized by the complete absorption of the remaining yolk sac, the opening of the mouth, and thus probably the main colonization of the gastrointestinal tract. Further, the swim-up fry must rise to the water’s surface to fill the swim bladder with air. The effective richness decreased significantly until SCF (Figure 1A). Microbial profiles of these samples displayed a strong heterogenicity, clustered remotely with no overlaps, and differed significantly in both groups (Figure 6C). Both stages’ BC displayed a similar number of families with an abundance stronger than 0.5% (SCF: 24 and SUF: 19), of which they shared over 50% of families (i.e., 13 families; Figure 7A). Of these families, an unknown family in the order Saprospirales was significantly higher in SCF (*p* ≤ 0.01), while the absence of Lachnospiraceae in SUF was also significant (*p* ≤ 0.05). At the molecular strain level, SCF’s BC consisted of 34 molecular strains with an abundance of at least 0.5%, while SUF’s BC consisted of only 22 zOTUs (Figure 7B). The two groups shared seven zOTUs (Figure 7A) and no significant differences on zOTU level were observed. The molecular strains zOTU 63 (*Aquirhabdus parva* with 97.5% similarity), zOTU 114 (*Rhizobacter profundi* with 98.4% similarity), zOTU 119 (*Agitococcus lubricus* with 96.5% similarity), zOTU 161 (*Pseudomonas atagonensis* with 99.5% similarity), and zOTU 175 (*Nevskia ramosa* with 97.9% similarity) were only observed in SCF.

We then compared SUF with FY3, as the time frame of two weeks between sampling of those stages was most comparable to the other time frames in the early developmental stages. Further, subsequent fry stages’ BC seemed to change in a non-physiological way, thus direct comparison was omitted. FY3 stage is a fry stage that had already been start-feed on 67 dpf and is thus already influenced by the feed’s impacts for 15 days. Between SUF and FY3, bacterial diversity increased, however, not significantly (Figure 1). Both stages’ samples displayed a strong heterogenicity in their ecological profiles and seemingly overlapped for some samples. Nevertheless, the two groups differed significantly in their microbial profiles (Figure 6D). SUF and FY3 stages showed nearly the same number of families; 19 and 18 families in SUF and FY3, respectively, and shared nine families (Figure 7A). Two families that were not shared between these stages were statistically significant, Clostridiaceae (*p* ≤ 0.05) and Vibrionaceae (*p* ≤ 0.05). The shared families, Flavobacteriaceae (*p* ≤ 0.05), Pseudomonadaceae (*p* ≤ 0.05), Rhodospirillaceae (*p* ≤ 0.05), and Streptococcaceae (*p* ≤ 0.05) displayed significant differences. The BC of SUF enclosed 22 zOTUs and the BC of FY3 26 zOTUs, whereby both stages shared five zOTUs (Figure 7B). Of these shared molecular strains, zOTU 14 (*Paraperlucidibaca baekdonensis* with 98.6% similarity), zOTU 18 (*Flavobacterium* species with 98.4% similarity) (Figure 8), zOTU 30 (*Aquirhabdus parva* with 100% similarity), and zOTU 77 (*Agitococcus* species with 97.3% similarity) were significantly different. The absence of zOTU 46 (*Ligilactobacillus salivarius* with 100% similarity), zOTU 51 (*Weissella cibaria* with 100% similarity), zOTU 70 (*Photobacterium phosphoreum* with 100% similarity), and zOTU 157 (*Ligilactobacillus salivarius* with 99.77% similarity) in one of these stages’ BC was statistically significant.

### 3.3. Bacterial Communities (BC) of the Late Developmental Stages from Swim-Up Fry to Fry

**Figure 9:**
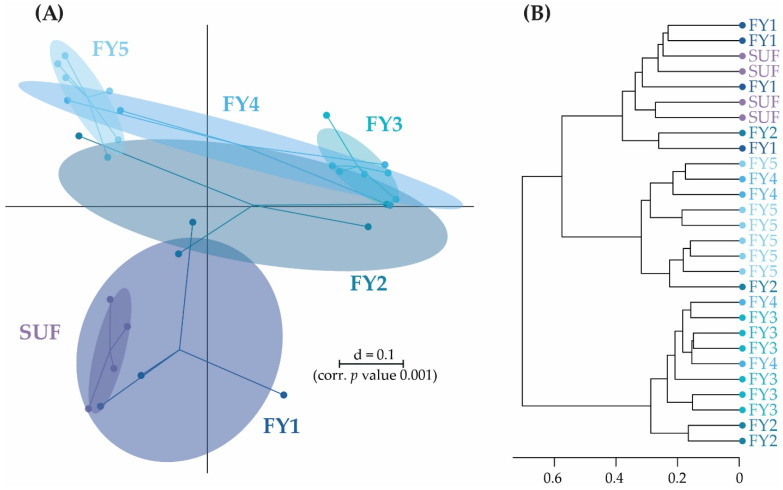
(**A**) β-diversity of late developmental stages’ BC is shown as a multidimensional-scaling (MDS) plot. The scale is an indicator for the variation in the taxonomic makeup of microbiota between samples (β-diversity) based on generalized UniFrac distances (d = 0.1 marks a 10% difference). (**B**) Dendrogram of samples from early developmental stages based on wards clustering method (d = 0.1 marks a 10% difference). SUF, swim-up fry; FY1, fry 69 dpf; FY2, fry 74 dpf; FY3, fry 82 dpf; FY4, fry 88 dpf; FY5, fry 95 dpf; dpf, days post fertilization.

**Figure 10:**
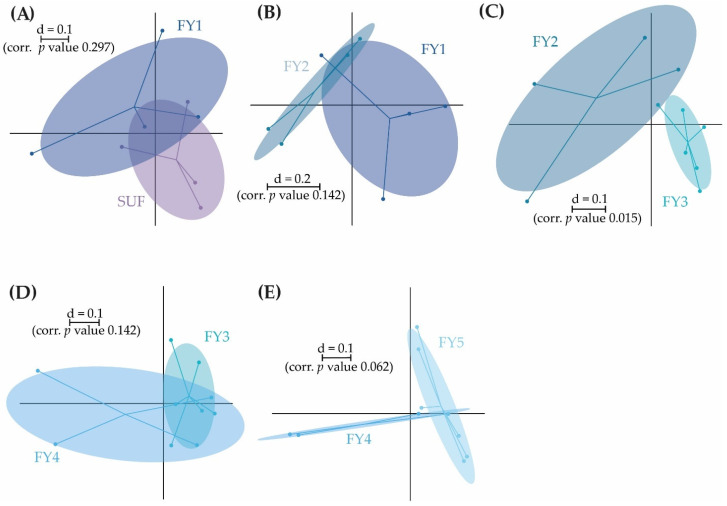
Pairwise β-diversity of the fry stages and SUF displayed as a multidimensional-scaling (MDS) plots. The scale is an indicator for the differences in the taxonomic makeup of microbiota between samples (β-diversity) based on generalized UniFrac distances (d = 0.1 marks a 10% difference). Pairwise β-diversity between SUF and FY1 (**A**); FY1 and FY2 (**B**); FY2 and FY3 (**C**); FY3 and FY4 (**D**); FY4 and FY5 (**E**). SUF, swim-up fry; FY1, fry 69 dpf; FY2, fry 74 dpf; FY3, fry 82 dpf; FY4, fry 88 dpf; FY5, fry 95 dpf; dpf, days post fertilization.

The following stages are fry stages (FY1–FY5; 69–95 dpf) that had been start-fed on 67 dpf. Here, line B provided insufficient sample numbers, thus, the following fry stages presented were derived only from line A. In these stages, only moderate differences in the BC between the subsequent stages were observed. The fry stages displayed fewer distinct shifts in their BC than the early developmental stages and higher consistency among the different stages (Figure 9 and Figure 10). All late developmental stages shared five families: Comamonadaceae, Moraxellaceae, Flavobacteriaceae, Alteromonadaceae, and Chitinophagaceae. In addition, five molecular strains occurred in all fry stages (zOTU 2, *Perlucidibaca piscinae* with 100% similarity; zOTU 4, *Flavobacterium* species with 95.84% similarity; zOTU 14, *Paraperlucidibaca baekdonensis* with 98.6% similarity; zOTU 27, *Curvibacter* species with 99.3% similarity; and zOTU 38, Chitinophagaceae with 95.84% similarity). The BC of the later developmental stages displayed fewer distinct clusters than the stages from EGG to SUF (Figure 2). SUF and FY1 BC were separated only by a time interval of two days (i.e., 67 dpf and 69 dpf) and feeding started on 67 dpf after the sampling. These two groups clustered variously on a separate branch of the sample similarity tree (Figure 9B). FY3 and FY5 samples split on different branches (Figure 9B; lower two branches). While FY2 samples were present in all three branches, FY4 samples split between lower two branches (Figure 9B).

For the early developmental stages, the time frame between sampling was around 14 days, based on the duration of the different developmental stages. Allowing a better comparison of the data, differences between late developmental stages were first displayed for every second sampling timepoint

**Figure 11:**
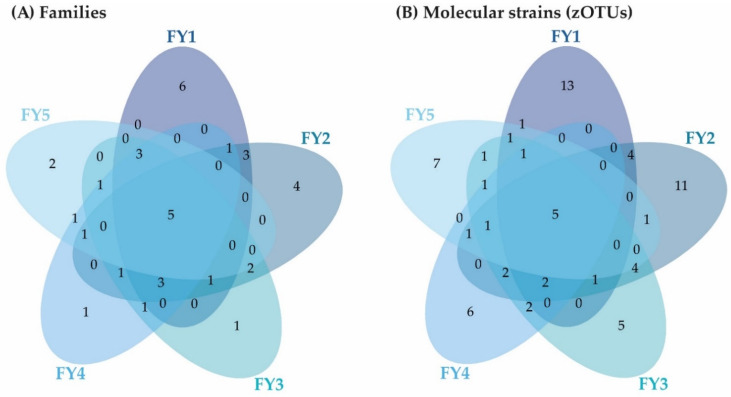
Venn diagram depicting unique and shared families (**A**) and molecular strains (zOTUs) (**B**) among the fry stages (FY1–FY5; cut-off: 0.5% mean relative abundance). FY1, fry 69 dpf; FY2, fry 74 dpf; FY3, fry 82 dpf; FY4, fry 88 dpf; FY5, fry 95 dpf; dpf, days post fertilization.

The effective richness and effective Shannon diversity increased between FY1 and FY3. Yet, this increase was not significant (Figure 1). There was no significant difference in the ecological profiles between FY1 and FY3 (*p* = 0.13), and both groups’ clusters apparently overlapped. On the dendrogram, the two stages clustered on different branches (Figure 9B). While FY1 was dominated by the families Rubritaleaceae, Comamonadaceae, and Pseudomonadaceae, FY3 showed higher abundances of Moraxellaceae, Lactobacillaceae, and Flavobacteriaceae. The family Rubritaleaceae vanished completely in FY3. In total, FY1 was composed of 22 families with an abundance above 0.5%, and FY3 of 18 families. Both stages shared twelve families (Figure 11A) and the absence of the family Clostridiaceae (*p* ≤ 0.05) in FY1 was significant. At the molecular strain level, FY1 was composed of 28 zOTUs with an abundance above 0.5% and FY3 of 26 zOTUs. Both stages shared ten zOTUs (Figure 11B). Significant differences at the molecular strain level have been observed for zOTU 46 (*Ligilactobacillus salivarius* with 100% similarity), zOTU 70 (*Photobacterium phosphoreum* with 100% similarity), and zOTU 157 (*Ligilactobacillus salivarius* with 99.8% similarity).

**Figure 12:**
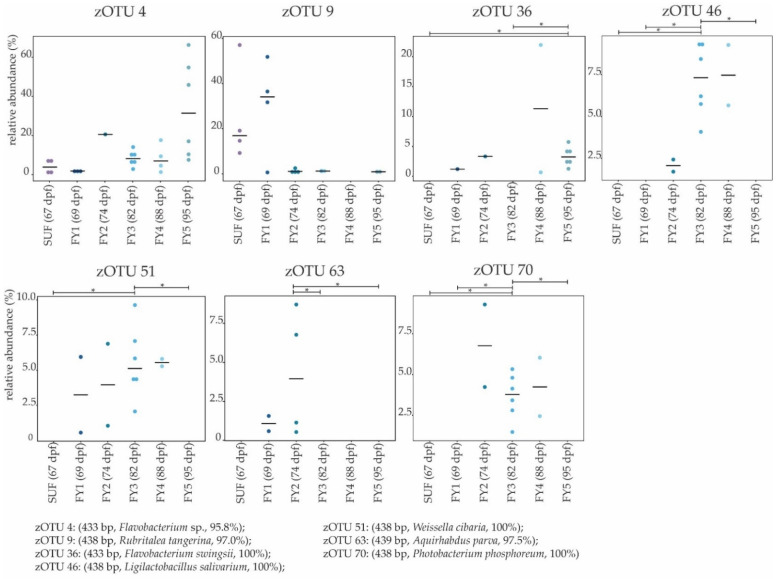
Molecular strains in the fry stages (FY1–FY5) and SUF; Boxplots depict the median (thick bar) and individual samples (points). Brackets above the individual boxplots indicate the significance level for pairwise comparison. zOTUs were identified by EzBiocloud; the sequence length, the closest relative taxon, and the sequence identity score of zOTUs are shown in the order of appearance. SUF, swim-up fry; FY1, fry 69 dpf; FY2, fry 74 dpf; FY3, fry 82 dpf; FY4, fry 88 dpf; FY5, fry 95 dpf; dpf, days post fertilization. *P* value summary: * *p* ≤ 0.05.

FY3 displayed a high effective richness, while a strong drop in abundance occurred thereafter. We further describe changes between these two stages. Comparing FY3 and FY5, the effective richness and Shannon effective diversity differed significantly (Figure 1). The ecological profiles of both stages’ BC showed a small overlap and differed significantly (*p* ≤ 0.05). On the dendrogram, the two stages clustered on different branches (Figure 9B). As described above, FY3 was dominated by the families Moraxellaceae, Flavobacteriaceae, and Lactobacillaceae, in descending order, while FY5 was dominated by the families Flavobacteriaceae, Moraxellaceae, and Chromobacteriaceae (Figure 4). FY3 consisted of 18 families, whereas FY5 consisted of only 13 families. Both stages shared nine families with an abundance above 0.5% (Figure 11A). The absence was significant for Clostridiaceae (*p* ≤ 0.05), Lactobacillaceae (*p* ≤ 0.05), Streptococcaceae (*p* ≤ 0.05), and Vibrionaceae (*p* ≤ 0.05), while Rhodospirillaceae was significantly lower in FY3 (*p* ≤ 0.05). In total, 26 zOTUs with a relative abundance above 0.5% were observed for FY3 and for FY5 a total of 20 zOTUs were observed (Figure 11B). At the molecular strain level, the absence of a zOTU in either FY3 or FY5 was significant for zOTU 36 (*Flavobacterium swingsii* with 100% similarity), zOTU 46 (*Ligilactobacillus salivarius* with 100% similarity), zOTU 51 (*Weissella cibaria* with 100% similarity), zOTU 70 (*Photobacterium phosphoreum* with 100% similarity), and zOTU 157 (*Ligilactobacillus salivarius* with 99.8% similarity; Figure 12).

In the following paragraphs, the time interval between sampling time points will be reduced again, comparing every stage with its successor. After FY1 differences occur, we only regard the growth and maturation of the fish.

When comparing the latest early developmental stage (i.e., SUF) with the following stage that is already part of the late developmental stages (i.e., FY1), bacterial diversity remained almost constant (Figure 1). The ecological profiles of these two stages were similar and did not differ significantly. The clusters of SUF and FY1 overlapped in half of the samples (Figure 10A). This close proximity can be seen exceptionally well on the dendrogram where all of these stages’ samples are clustered on one branch (Figure 9B). SUF’s BC consisted of 19 families with an abundance higher than 0.5%, while FY1’s BC contained 22 families (Figure 11A). The absence of Rhodospirillaceae in SUF was significant (*p* ≤ 0.05). SUF’s BC consisted of 22 zOTUs, while FY1’s BC of 28 zOTUs (Figure 11B). No significant differences at the zOTU level were observed.

Between FY1 and FY2 BC, bacterial diversity increased without differing significantly (Figure 1). The samples of these two stages’ ecological profiles displayed overlapping clusters and were not significantly different (Figure 10B). Nevertheless, they clustered on different branches of the dendrogram (Figure 9B). In the Venn diagram, the families and the molecular strains (zOTUs) that were unique for one stage or shared among two or more developmental stages are shown (Figure 11A,B). At the family level, FY1’s BC consisted of 22 families and FY2’s BC of 21 families with a relative abundance of 0.5% or higher. The two stages’ BC shared 13 families (Figure 11A). None of the families observed differed significantly between FY1 and FY2. FY1 contained 28 zOTUs, while FY2 32 zOTUs (Figure 11B). Both stages’ BC shared 12 molecular strains, while no zOTU differed significantly.

Between the BC of FY2 and FY3, the effective richness was comparable (Figure 1A), while the microbial profiles differed significantly (Figure 9C). The BC of the stages FY2 and FY3 contained 21 and 18 families, respectively, and shared 14 families (Figure 11A). No families differed significantly between those two stages. At the molecular strain level, FY2 consisted of 32 zOTUs and FY3 consisted of 26 zOTUs (Figure 11B). These two stages shared 15 zOTUs, however, the absence of zOTU 63 (*Aquirhabdus parva* with 97.5% similarity) in FY3 and the absence of zOTU 157 (*Ligilactobacillus salivarius* with 99.8% similarity) in FY2 was significant (*p* ≤ 0.05; Figure 12).

When comparing FY3 with FY4, a subtle decrease of the bacterial diversity was observed (Figure 1). The two groups displayed overlapping clusters in the multidimensional-scaling plot (Figure 10D) and the proximity of overlapping samples could be seen exceptionally well in the dendrogram (Figure 9B). Hence, β-diversity displayed no significant difference (Figure 10D). FY3 still contained 18 families as did FY4, of which the two groups shared 15 families (Figure 11A). FY3 contained 26 and FY4 21 zOTUs, of which both groups shared 14 zOTUs (Figure 11B). No significant differences have been observed at either family or zOTU level.

Looking at FY4 and FY5, α-diversity displayed no significant difference. Nonetheless, effective richness and Shannon effective diversity decreased (Figure 1). The ecological profiles of these two groups displayed no significant difference (*p* = 0.062; Figure 10D), overlapped slightly; while half of the FY4 samples clustered on another branch than FY5 samples (Figure 9B). In general, FY5 samples seemed very homogenous in both the multidimensional-scaling plot and the dendrogram (Figure 9). While FY4 still had 18 families, the count decreased to 13 families in FY5 BC (Figure 11A). Both stages shared eleven families. The growing difference between the two stages was observed in the diversity (Figure 10E) and the decrease of shared families to only eleven counts (Figure 11A). At zOTU level, FY4’s BC consisted of 21, while FY5’s BC of 20 zOTUs, of which they shared nine zOTUs (Figure 11B). No significant differences were found between families and zOTU.

Summarizing all results, the changes during early development were larger than the changes during the late developmental stages. The richness varied between the BC of stages EGG, EYG, and SCF, then increased until FY3 and dropped until the last sampling in FY5. β-diversity changed significantly in successor stages in the early development, while in the late developmental stages no significance differences occurred, with the exception of FY2 to FY3. All stages’ BC were dominated by Pseudomonadota at the phylum level. In EGG, several families displayed similarly high abundances, while the later developmental stages were dominated by one or two families each. However, these families changed over time, with Moraxellaceae being the family that dominated most stages’ BC.

## 4. Discussion

In the present study, we investigated the ontogenetic development in the BC of brown trout in different developmental stages from unfertilized egg to fry. To the best of our knowledge, this is the first study using 16S gene amplicon rRNA sequencing for assessing the microbiota in brown trout during ontogeny. The study was conducted in a fish farm run as a commercial aquaculture, to provide a realistic representation of the BC under commercial farming conditions. The handling in a commercial fish farm seemed to have no influence on data quality and especially no contamination has been detected in the samples. The sampling of the complete fish resulted in a high amount of host DNA during DNA extraction and library preparation, and probably subsequent partial inhibition of the end product in several samples, thus some single samples had to be excluded.

Results from this work showed that during early development (i.e., 0 to 67 dpf) the BC of the complete fish changed strongly between the stages regarding the effective richness, family, and even phylum composition. These strong shifts are consistent with other studies in salmonids [27,59]. Nevertheless, there is a lack of knowledge regarding the ontogenetic development of different fish species and its influence on the fish’s health. Similar to the findings in the brown trout’s eggs sampled in this study (i.e., EGG and EYG), other authors described a diverse BC [60,61] dominated by the phylum Pseudomonadota in the embryonal stages [18,27,62,63]. Wilkins et al. (2016) [18] showed that in brown trout eggs’ BC, the maternal influence was negligible, whereas the fraternal impact and bacterial nutrient availability displayed the strongest effects. The high abundance of Lachnospiraceae in the unfertilized egg found in our study has not been described elsewhere, although most other studies described fertilized eggs only. Interestingly, we found a lower bacterial diversity in the EYG stage compared to the EGG stage. Wilkins et al. (2015) reported a decrease in α-diversity from early to late embryos in whitefish (*Coregonus* species) whereby the eggs were fertilized and the eyed eggs were sampled at a later time point than in the present study [64]. It has been suggested that the surface of eggs is rich in bacterial substrate [25,26,65], therefore, bacteria might colonize the early chorion stages in higher abundances, while bacterial diversity decreases in later egg stages as nutrients on the egg surface decrease [66]. Whether the diversity decreased until the late embryonic stage and increased immediately in the SCF stage remains unknown due to the lack of sampling just before hatching. When eggs were fertilized and started developing (i.e., EYG 35 dpf), the ecological profiles differed strongly from all other samples (Figure 8 and Figure 10). Even though different spawners and milters were combined, the variations between different parent animals could not be traced at 35 dpf and all EYG samples showed a similar BC that strongly differed from all other tested stages. In fertilized eggs from channel catfish (*Ictalurus punctatus*), brown trout, and ballan wrasse (*Labrus bergylta*), the BC was described to be influenced by the holding tanks, genetics, and egg disinfection [18,62,67]. Furthermore, for European seabass (*Dicentrarchus labrax*) and gilthead seabream (*Sparus aurata*), an influence of sampling site, species, and sampling type on the BC of eggs and water have been described [63]. In a study by Najafpour et al. (2021) [63], gilthead seabream’s BC from a certain sampling place displayed high abundances of the genus *Psychrobium*, while for European seabrass’s a high abundances of the genus *Cyanobacteria* was typical for a certain location. Other studies described Flavobacteriacae as a site-specific family [18,62,63], while Rhodobacteraceae is suggested to be an important environmental-associated family that is found often on fertilized eggs [63,68,69]. In the present study, the most common families in EYG were Comamonadaceae, Sphingomonadaceae, Corynebacteraceae, and Flavobacteriaceae in descending order (Figure 4), and further, Comamonadaceae was mostly composed of the genera *Rhodoferax* (14.6%), *Hydrogenophaga* (9.6%), and *Paucibacter* (5.1%). At the molecular strain level, in descending order, the most abundant strains of these genera were zOTU 24 (*Paucibacter oligotrophus* with 98.6% similarity), associated with freshwater [70], zOTU 20 and 23 (both *Hydrogenophaga taeniospiralis* with 99.8% and 100% similarity, respectively), which has been first isolated from soil [71], and zOTU 16 (*Rhodoferax bucti* with 98.6% similarity), isolated from freshwater [72]. This finding supports the theory of an environmentally associated BC. Further, the family Sphingomonadaceae (mostly genus *Chakrabartia*; 19.2%) with its molecular strain zOTU 3 (*Chakrabartia* species with 100% similarity to Cr-cl17 (DQ295907)) is most probably associated with water, as the strain Cr-cl17 has been isolated from backwash water in Germany [73]. Comparison of embryonal stages’ BC to different studies is hardly possible, as early life BCs in fish are suggested to be species and stage-specific [74]. In general, knowledge of the BC in early developmental stages and even before the hatching is important as shifts or differences in the BC in these embryonal stages might be associated with higher embryonal mortality and a longer time until hatching [18], however, this association has not been tested in this work.

The hatching of the sac fry larvae exposed the fish and the mucosal organs for the first time to the environment and its BC [75,76,77]. This encounter and the following colonization of the sac fry with microorganisms might play an important role in the development of mucosal organs and the immune system of the fish [78,79,80]. Further, prior to hatching, oxygen is taken up by cutaneous breathing, which changes with hatching to pharyngeal oxygen uptake and could affect the BC [81]. Compared to the previous EYG stage, the SCF stage displayed a vastly different BC with the highest abundance of all sampled stages composed of the families Moraxellaceae, Comamonadaceae, Burkholderiaceae, and Nevskiaceae, in descending order. A strong difference between eggs and hatched larvae has been described by other authors as well [27]. Additionally, the higher richness of pre-feeding larvae and the strongest difference to most other stages has been described before [82]. Nikouli et al. (2019) [83] described sac fry stages’ BC to be highly comparable to the BC of the rearing water. This might arise from osmoregulation processes in larvae before mouth opening [74,84]. In this work, SCF stages have been dominated by the molecular strains zOTU 2 (*Perlucidibaca piscinae* with 99.5% similarity), associated with freshwater [85], and zOTU 49 (*Limnobacter thiooxidans* with 100% similarity) associated with freshwater lake sediment from the Chiemsee in Germany [86]. Further dominant strains were zOTU 27 (*Curvibacter* species with 99.3% similarity) with the top hit strain PAE-UM (accession KQ483358) that has been isolated from river sediment and zOTU 119 (*Agitococcus lubricus* with 96.5% similarity) isolated from fresh water [87]. These findings not only support the work from Nikouli et al. (2019) [83] but further support the theory that BC from sac fry larvae is associated with the surrounding water and environment.

When all nutrients in the yolk sack are consumed, the fish starts orally consuming feed. In this study, the swim-up fry stage (i.e., SUF) was sampled before first feeding. Therefore, in this stage, feed and feed-associated microbiota have not yet influenced the fry. However, even though the SUF has not been start-fed and no changes in the environment occurred, we currently assume that changes might be either due to genetic factors, development [88], the feeding on environmental bacteria, or egg residuals [89,90,91]. Such a strong change in BC from sac fry stages until start-feeding has been described as well by Bakke et al. (2015) [92]. As in the SCF stage, the BC is dominated by the molecular strain zOTU 2 (*Perlucidibaca piscinae* with 99.5% similarity). However, further high abundant zOTUs belonged to the genera *Rubritalea*, *Pseudomonas*, or *Aeromonas*. zOTU 9 (*Rubritalea tangerine* with 97.0% similarity) has been isolated from marine animals [93], while zOTU 7 (*Aeromonas* species) displayed a 100% similarity with seven possible strains. It should be noted that some *Aeromonas* species are considered pathogenic [94,95]. zOTU 5 (*Pseudomonas gessardii* with 100% similarity) and zOTU 28 (*Pseudomonas proteolytica* with 100% similarity) are associated with mineral water [96] and cyanobacteria [97], respectively.

In the late developmental stages (i.e., 69 to 95 dpf), the effective richness increased first, then dropped after reaching the highest effective richness in FY3 stage. There are only small changes between single stages, although the shifts between FY1, FY3, and FY5 are larger. Most studies dealing with later developmental stages only analyzed the gut microbiome, as the gut microbiome is presumed to have a major influence on the immune defense and digestive system of the fish [98]. However, the microbiome on the surface of the fish and in the mucous has a protective effect against pathogens [99,100], thus the BC of the complete fish is relevant. Further, in some studies, samples were collected from the complete organism for the early developmental stages and only from the intestine for later developmental stages [27,101,102], hampering a relevant comparison of these stages due to biased sampling. Other studies only considered either the egg [62,103] or the later developmental stages after hatching [104]. In order to collect comparable data over a longer period of development, it is necessary to bring together all samples in succession using exactly the same methods of sampling, DNA extraction, library preparation, sequencing, and even data analysis [105]. Consequently, it is only possible to compare the results of different microbiome studies to a very limited extent, as the impact of the chosen approach is high. In the present study, for the first time various developmental stages of brown trout from the same fish hatchery have been sampled and processed in the same manner.

Between the stages SUF and FY1, start-feeding had been initiated. Hence, FY1-fish’s BC had been influenced by feed-specific bacteria. Even if SUF had not been in contact with feed yet, the family Rubritaleaceae, which has been associated in other studies with feeding [106], appeared and increased in number in FY1 stage. Interestingly, in later stages, this family seemed to have vanished (Figure 4), while in ballan wrasse, Rubritaleaceae have been mostly associated with early embryogenic BC [62]. Remarkably, the genus *Perlucidibaca* decreased between SUF (19.8%) and FY1 (1.2%), but increased again later (FY2, 11.4%; FY3, 16.0%; FY4, 29.0%). In the last fry stage (i.e., FY5) it decreased slightly (15.9%). In difference to the other fry stages, SUF and FY1 were dominated mostly by the genera *Rubritalea* and *Pseudomonas*, while later stages beside FY5 displayed *Flavobacterium*, *Paraperlucidibaca*, *Perlucidibaca*, and *Photobacterium* for genera with more than 2.0% of relative abundance. While in other studies *Flavobacterium* [27,63,103] and *Photobacterium* [27,63,101] were often described in fish, the biological origin of the other genera is harder to place.

Surprisingly, after the FY3 stage, the bacterial diversity decreased (Figure 1) and in FY5, the bacterial community displayed strong changes compared to the other stages. In comparison to all other stages, this stage was dominated by the genus *Flavobacterium* (33.5%) followed by *Deefgea* (18.5%), a genus not displayed in higher abundances in any other stage. Both genera include species that are known or at least thought to be pathogenic to fish [107]. While *Flavobacterium branchiophilum*, *Flavobacterium columnare*, and *Flavobacterium psychrophilum* and other species are known fish pathogens [108], other *Flavobacterium* species are not. In this study, three different molecular strains, zOTU 4, zOTU 18 and zOTU 22, which all belonged to the genus *Flavobacterium*, displayed high abundances in the early developmental stages and were significantly different between the developmental stages. While zOTU 4 and zOTU 18 first appeared in SCF and SUF and showed high abundances especially later in FY3, zOTU 22 occurred almost exclusively in the EYG stage and was found in every sample of both lines. zOTU 4 occurred in all later developmental stages with the highest abundance in the fry stage FY5. It was not possible to determine in this study whether certain zOTUs were pathogenic, as 16S rRNA gene amplicon sequencing allows no direct identification of species but a comparison of strain similarity. Nevertheless, the large increase in *Flavobacterium* species at the end of the experiment indicates infection with a pathogenic species. Even though the *Flavobacterium* and probable *Deefgea* species found here did not display direct pathogenicity with clinical signs, such bacteria might cause a loss of fitness in fry, for instance causing subsequent mass infection with *Ichthyobodo necator* after 95 dpf. Dysbiosis of the microbiome increases the susceptibility of the fish to other pathogens. Furthermore, it has already been shown that infection with parasites, such as *Ichthyophthirius multifillis*, may decrease bacterial diversity and an increase in Flavobacteriaceae in fish [109].

The data presented suggest that the manipulation of the fish’s BC might be possible directly after oviposition and hatching. Because these two timeframes display great shifts in the BC, the introduction of beneficial strains might be best possible in those stages. However, currently, no suitable probiotic strain has been identified. In future studies, it is advisable to repeat the sampling of brown trout during ontogeny with higher sample numbers and if possible, even in different fish farms using the exact same approach (i.e., sampling *in toto*, using the same DNA extraction, library preparation, and sequencing) since otherwise comparison is restricted [105].

## 5. Conclusions

The present study describes the BC from egg pre-fertilization to fry 95 dpf. We found that brown trout’s BC changed strongly during early development and then stabilized after the first feeding. The later developmental stages displayed considerably less significant differences between the stages BC. Since hardly any studies exist that sampled all mentioned stages with the same method and coherent data for the brown trout’s ontogeny are missing, this study provides a new insight into the development of brown trout BC during ontogeny. Further, opportunities for an intervention (e.g., probiotic, prebiotic, or optimized rearing condition) in the BC and thus, the fitness of fish, might be possible directly after fertilizing the egg and shortly after hatching. It is likely, as other authors described as well, that an intervention in the BC of brown trout during development needs to be adjusted to the rearing environment. In sum, even a more sophisticated and detailed analysis of the BC in embryogenic stages and further methods such as metagenomics will be needed to understand the colonization of brown trout embryos.

## Figures and Tables

**Table 1:** Read count and α-diversity (effective richness) as average mean.

Sample	Read Count	Effective Richness
EGG (0 dpf)	20,392	51.2
EYG (35 dpf)	19,153	28.2
SCF (48 dpf)	3818	59.2
SUF (67 dpf)	12,610	28.2
FY1 (69 dpf)	6550	38.8
FY2 (74 dpf)	13,727	48.5
FY3 (82 dpf)	5720	48.9
FY4 (88 dpf)	8264	39.0
FY5 (95 dpf)	11,846	28.0

EGG, egg; EYG, eyed egg; SCF, sac fry; SUF, swim-up fry; FY, fry; dpf, days post fertilization.

## Data Availability

The data that support the findings of this study are openly available in the Sequence Read Archive under the reference number PRJNA909450.

## Supplementary Materials

The following supporting information can be downloaded at: https://www.mdpi.com/article/10.3390/microorganisms11010211/s1, Table S1: OTU Table of all samples, Table S2: zOTU Table of all samples.
